# BEHRT: Transformer for Electronic Health Records

**DOI:** 10.1038/s41598-020-62922-y

**Published:** 2020-04-28

**Authors:** Yikuan Li, Shishir Rao, José Roberto Ayala Solares, Abdelaali Hassaine, Rema Ramakrishnan, Dexter Canoy, Yajie Zhu, Kazem Rahimi, Gholamreza Salimi-Khorshidi

**Affiliations:** Deep Medicine, Oxford Martin School, University of Oxford, Oxford, United Kingdom

**Keywords:** Preventive medicine, Experimental models of disease

## Abstract

Today, despite decades of developments in medicine and the growing interest in precision healthcare, vast majority of diagnoses happen once patients begin to show noticeable signs of illness. Early indication and detection of diseases, however, can provide patients and carers with the chance of early intervention, better disease management, and efficient allocation of healthcare resources. The latest developments in machine learning (including deep learning) provides a great opportunity to address this unmet need. In this study, we introduce BEHRT: A deep neural sequence transduction model for electronic health records (EHR), capable of simultaneously predicting the likelihood of 301 conditions in one’s future visits. When trained and evaluated on the data from nearly 1.6 million individuals, BEHRT shows a striking improvement of 8.0–13.2% (in terms of average precision scores for different tasks), over the existing state-of-the-art deep EHR models. In addition to its scalability and superior accuracy, BEHRT enables personalised interpretation of its predictions; its flexible architecture enables it to incorporate multiple heterogeneous concepts (e.g., diagnosis, medication, measurements, and more) to further improve the accuracy of its predictions; its (pre-)training results in disease and patient representations can be useful for future studies (i.e., transfer learning).

## Introduction

The field of precision healthcare aims to improve the provision of care through precise and personalised prediction, prevention, and intervention. In recent years, advances in deep learning (DL) - a subfield of machine learning (ML) - has led to great progress towards personalised predictions in cardiovascular medicine, radiology, neurology, dermatology, ophthalmology, and pathology. For instance, Ardila *et al.*^1^ introduced a DL model that can predict the risk of lung cancer from a patient’s tomography images with 94.4% accuracy; Poplin *et al.*^2^ showed that DL can predict a range of cardiovascular risk factors from just a retinal fundus photograph, and more examples can be found in other works^3,4^. A key contributing factor to this success, in addition to the developments in DL algorithms, was the massive influx of large multimodal biomedical data, including but not limited to, electronic health records (EHR)^5^.

The adoption of EHR systems has greatly increased in recent years; hospitals that have adopted EHR systems now exceeds 84% and 94% in the US and UK, respectively^6,7^. As a result, EHR systems of a national (and/or a large) medical organisation now are likely to capture data from millions of individuals over many years. Each individual’s EHR can link data from many sources (e.g., primary and hospital care) and hence contain “concepts” such as diagnoses, interventions, lab tests, clinical narratives, and more. Each instance of a concept can mean a single or multiple data points. Just a single hospitalisation, for instance, can generate thousands of data points for an individual, whereas a diagnosis can be a single data point (i.e., a disease code). This makes large-scale EHR a uniquely rich source of insight and an unrivalled data for training data-hungry ML models.

In traditional research on EHR data (including the ones using ML), individuals are represented by models as a vector of attributes, or “features”^8^. This approach relies on experts’ ability to define the appropriate features and design the model’s structure (i.e., answering questions such as “what are the key features for this prediction?” or, “which features should have interactions with one another?”). Recent developments in deep learning, however, provided us with models that can learn useful representations (e.g., of individuals or concepts) from raw or minimally-processed data, with minimal need for expert guidance^9^. This happens through a sequence of layers, each employing a large number of simple linear and nonlinear transformations to map their corresponding inputs to a representation; this progress across layers results in a final representation in which the data points form distinguishable patterns.

Such properties of DL models and their success in a wide range of applications led to their growing popularity in EHR research. One of the earliest works in applying deep learning to EHR, Liang *et al*.^10^ showed that deep neural networks can outperform support vector machines (SVM) and decision trees paired with manual feature engineering, over a number of prediction tasks on a number of different datasets. Tran *et al*.^11^ proposed the use of restricted Boltzmann machines (RBM) for learning a distributed representation of EHR, which was shown to outperform the manual feature extraction, when predicting the risk of suicide from individuals’ EHR. In a similar approach, Miotto *et al*.^12^ employed a stack of denoising autoencoders (SDA) instead of RBM, and showed that it outperforms many popular feature extraction and feature transformation approaches (e.g., PCA, ICA^13^ and Gaussian mixture models) for providing classifiers with useful patient representations to predict the onset of a number of diseases from EHR.

These early works on the application of DL to EHR did not take into account the subtleties of EHR data (e.g., the irregularity of the inter-visit intervals, and the temporal order or events). In an attempt to address this, Nguyen *et al*.^14^ introduced a convolutional neural network (CNN) model called Deepr (Deep record) for predicting the probability of readmission; they treated one’s medical history as a sequence of concepts (e.g., diagnosis and medication) and inserted a special word between each pair of consecutive visits to denote the time difference between them. In another similar attempt, Choi *et al*.^15^ introduced a shallow recurrent neural network (RNN) model to predict the diagnoses and medications that are likely to occur in the subsequent visit. Both these works employed some embedding techniques to map non-numeric medical concepts to an algebraic space in which the sequence models can operate.

One of the improvements that was next introduced to the DL models of EHR aimed to enable them to capture the long-term dependencies among events (e.g., key diagnoses such as diabetes can stay a risk factor over a person’s life, even decades after their first occurrence; certain surgeries may prohibit certain future interventions). Pham *et al*.^16^ introduced a Long Short-Term Memory (LSTM) architecture with attention mechanism, called DeepCare, which outperformed standard ML techniques, plain LSTM, and plain RNN in tasks such as prediction of the onset of diabetes. In a similar development, Choi *et al*.^17^ proposed a model based on reverse-time attention mechanism to consolidate past influential visits using an end-to-end RNN model named RETAIN for the prediction of heart failure. RETAIN outperformed most of the models at the time of its publication and provided a decent baseline for the EHR learning research.

Given the success of deep sequence models and attention mechanisms in the past DL research for EHR, we aim to build on some of the latest developments in deep learning and natural language processing (NLP)– more specifically, Transformer architecture^18^ – while taking into account various EHR-specific challenges, and provide improved accuracy for the prediction of future diagnoses. We named our model BEHRT (i.e., BERT for EHR), due to architectural similarities that it has with (and our original inspirations that came from) BERT^18^, one of the most powerful Transformer-based architectures in NLP.

## Methods

### Data

In this study, we used Clinical Practice Research Datalink (CPRD)^19^: it contains longitudinal primary care data from a network of 674 GP (general practitioner) practices in the UK, which is linked to secondary care (i.e., hospital episode statistics or HES) and other health and administrative databases (e.g., Office for National Statistics’ Death Registration). Around 1 in 10 GP practices (and nearly 7% of the population) in the UK contribute data to CPRD; it covers 35 million patients, among whom nearly 10 million are currently registered patients^19^. CPRD is broadly representative of the population by age, sex, and ethnicity. It has been extensively validated and is considered as the most comprehensive longitudinal primary care database^20^, with several large-scale epidemiological reports^19,21,22^ adding to its credibility.

HES, on the other hand, contains data on hospitalisations, outpatient visits, accident and emergency for all admissions to National Health Service (NHS) hospitals in England^23^. Approximately 75% of the CPRD GP practices in England (58% of all UK CPRD GP practices) participate in patient-level record linkage with HES, which is performed by the Health and Social Care Information Centre^24^. In this study, we only considered the data from GP practices that consented to (and hence have) record linkage with HES. The importance of primary care at the centre of the national health system in the UK, the additional linkages, and all the aforementioned properties, make CPRD one of the most suitable EHR datasets in the world for data-driven clinical/medical discovery and machine learning.

### Pre-processing of CPRD

**Figure 1:**
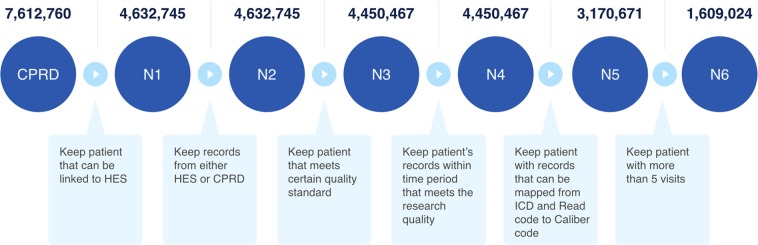
Linkage and filtering of CPRD data. This flow lists all the key steps of our data cleaning and linkage procedure. At each step, the number of patients that are included is shown. As you can see, we started with nearly 8 million patients and our final data (used for training and evaluation of our models) consists of 1.6 million patients, each meeting our inclusion criteria.

We start with 8 million patients; in our first filtering, we only included patients that are eligible for linkage to HES and meet CPRD’s quality standards (i.e., using the flags and time windows that CPRD provides to indicate the quality of one’s EHR). Furthermore, to only keep the patients that have enough history to be useful for prediction, we only kept individuals who have at least 5 visits in their EHR. At the end of this process, we are left with $P=1.6$ million patients to train and evaluate BEHRT on. More details on our inclusion/exclusion steps, and the number of patients after each one of them, can be seen in Fig. 1.

In CPRD, diseases are classified using Read code^25^ and 10th revision of the International Statistical Classification of Diseases and Related Health Problems (ICD-10)^26^, for primary and hospital care, respectively. In ICD-10, one can define diseases at the desired level of granularity that is appropriate for the analysis of interest, by simply choosing the level of hierarchy one wants to operate at; for instance, operating at ICD-10 chapter level will lead to 22 health categories, while operation at ICD-10 sub-chapter level will lead to over 1,900. After that, we mapped both ICD-10 codes (at level 4) and Read codes to Caliber codes^27^, which is an expert checked mapping dictionary from University College London. Eventually, this resulted in a total of $G=301$ codes for diagnoses. We denote the list of all these diseases as $D={\{{d}_{i}\}}_{i=1}^{G}$, where ${d}_{i}$ denotes the $i$th disease code.

**Figure 2:**
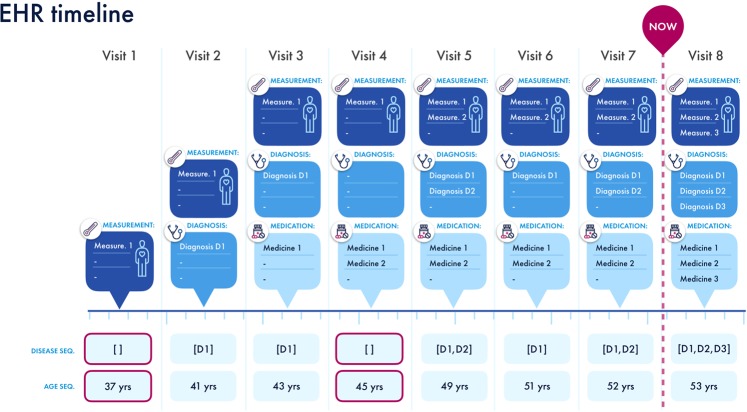
Preparation of CPRD data for BEHRT. An example patient’s EHR sequence can be seen in the figure, which consists of 8 visits. In each visit, the record can consist of concepts such as diagnoses, medications and measurements; all these values are artificial and for illustration purposes. For this study, we are only interested in age and diagnoses. Therefore, as shown in at the bottom of the figure, we have taken only the diagnosis and age subset of the record to form the necessary sequences. This resulting sequence is how we represent every patient’s EHR in our modelling process. Note that the visits shown in purple boxes are not going to be represented to the model, due to them lacking diagnoses.

In our final data, for each patient $p\in \{1,2,\ldots ,P\}$ the medical history consists of a sequence of visits to GP and hospitals; each visit can contain concepts such as diagnoses, medications, measurements and more. In this study, however, we are only considering the diagnoses; we denote each patient’s EHR as ${V}_{p}=\{{{\bf{v}}}_{p}^{1},{{\bf{v}}}_{p}^{2},{{\bf{v}}}_{p}^{3},\ldots ,{{\bf{v}}}_{p}^{{n}_{p}}\}$, where ${n}_{p}$ denotes the number of visits in patient $p$’s EHR, and ${{\bf{v}}}_{p}^{j}$ contains the diagnoses in the $j$th visit, which can be viewed as a list of ${m}_{p}^{j}$ diagnoses (i.e., ${{\bf{v}}}_{p}^{j}=\{{d}_{1},\ldots ,{d}_{{m}_{p}^{j}}\}$). In order to prepare the data for BEHRT, we order the visits (hence diseases) temporally, and introduce a term to denote the start of medical history (i.e., $CLS$), and the space between visits (i.e., $SEP$), which results in a new sequence, ${V}_{p}=\{CLS,{{\bf{v}}}_{p}^{1},SEP,{{\bf{v}}}_{p}^{2},SEP,\ldots ,{{\bf{v}}}_{p}^{{n}_{p}},SEP\}$, that from now on will be how we see/denote each patient’s EHR as. This process is illustrated in Fig. 2.

### BEHRT: A Transformer-based Model for EHR

In this study, we aim to use a given patient’s past EHR to predict his/her future diagnoses (if any), as a multi-label classification problem (i.e., simultaneously predicting a probability for each and every disease); this will result in a single predictive model that scales across a range of diseases (as opposed to needing to train one predictive model per disease). Modelling EHR sequences requires dealing with four key challenges^16^: (C.1) complex and nonlinear interactions among past, present and future concepts; (C.2) long-term dependencies among concepts (e.g., diseases occurring early in the history of a patient effecting events far later in future); (C.3) difficulties of representing multiple heterogeneous concepts of variable sizes and forms to the model; and (C.4) the irregular intervals between consecutive visits.

Similarities between sequences in EHR and natural language lead to successful transferability of techniques. For instance, techniques such as BoW^12^, Skip-gram^15^, RNN^17^, and attention^16,28^ (*a la* their NLP usage) are used for learning complex EHR representations. Also, Velupillai *et al*.^29^ also suggested to use NLP modelling for knowledge extraction, mental health treatment and large-scale clinical research from unstructured EHR. In addition, Huang *et al*.^30^ also proposed a NLP model (ClinicalBERT) to predict the 30-day hospital re-admission. In this study, we get our inspiration from the great success of Transformers^31^, and more specifically, a Transformer-based architecture known as BERT^18^. We refer readers to the original papers^18,31^ for an exhaustive background description for both Transformer and BERT.

**Figure 3:**
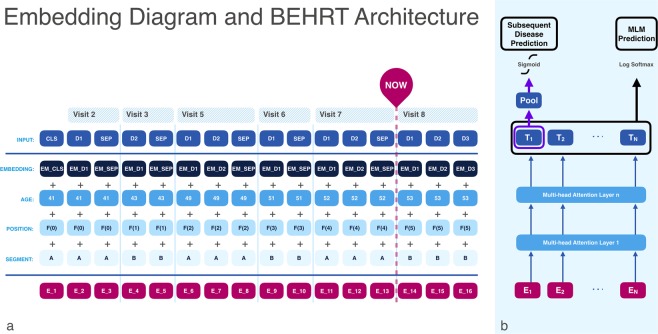
BEHRT architecture. Using the artificial data shown in Fig. 2, section (**a**) shows how BEHRT sees one’s EHR. In addition to diagnosis and age, BEHRT also employs an encoding for an event’s positions (shown as POSITION) and an encoding for visit (shown as SEGMENT with A and B), which alternates between visits. The summation of all these embeddings results in a final embedding shown at the bottom of (**a**), which will be the latent contextual representation of one’s EHR at a given visit’s diagnosis. Section (**b**) on the other hand shows BEHRT’s Transformer-based architecture. It is first pre-trained by the MLM task, to learn the network parameters (including the disease embeddings) that can predict the masked disease tokens. When training the model in downstream tasks (i.e., T1 to T3 - detailed explanation can be found in section: Disease Prediction), the model finetunes the weights pre-trained in the MLM task and learns the weights for the classification layer (i.e., mapping ${T}_{1}$ to the pooling layer and finally to the subsequent diseases classifier).

By depicting diagnoses as words, each visit as a sentence, and a patient’s entire medical history as a document, we facilitate the use of multi-head self-attention, positional encoding, and masked language model (MLM), for EHR – under a new architecture we call BEHRT. Figure 3b illustrates BEHRT’s architecture, which is designed to pre-train deep bidirectional representations of medical concepts by jointly conditioning on both left and right contexts in all layers. The pre-trained representations can be simply employed for a wide range of downstream tasks, e.g., prediction of the next diseases, and disease phenomapping. Such bidirectional contextual awareness of BEHRT’s representations is a big advantage when dealing with EHR data, where due to variabilities in individuals’ health as well as practice of care, or simply due to random events, the order at which certain diseases happen can be reversed, or the time interval between two diagnoses can be shorter or longer than actually recorded.

BEHRT has many structural advantages over many of the previous methods for modelling EHR data. Firstly, we use feed-forward neural networks to model the temporal evolution of EHR data through utilising various forms of sequential concepts in the data (e.g., age, order of visits), instead of using traditional state-of-the-art RNN and CNN that were explored in the past^14,17^. Recurrent neural networks are known to be notoriously hard to train due to their exploding and vanishing gradient problems^32^; these issues hamper these networks’ ability to learn (particularly, when dealing with long sequential data). On the other hand, convolutional neural networks only capture limited amount of information with convolutional kernels in the lower layers, and need to expand their receptive field through increasing the number of layers in a hierarchical architecture. BEHRT’s feed-forward structure alleviates the exploding and vanishing gradient problems and captures information by simultaneously considering the full sequence -- a more efficient training through learning the data in parallel rather than in sequence (unlike the RNN).

The embedding layer in BEHRT, as shown in Fig. 3, learns the evolution of one’s EHR through a combination of four embeddings: disease, “position”, age, and “visit segment” (or “segment”, for short). This combination enables BEHRT to define a representation that can capture one’s EHR in as much detail as possible. Disease codes are of course important in informing the model about the past diagnoses in one’s EHR. That is, there are many common disease trajectories and morbidity patterns^33^ that knowing one’s past diseases can improve the accuracy of the prediction for their future diagnoses. Positional encodings determine the relative position of concepts in EHR sequence, and enable the network to capture the positional interactions among diseases. In this paper, we used a pre-determined encoding (as proposed by Vaswani *et al.*^31^), to avoid weak learning of positional embedding caused by imbalanced distribution of medical sequence length in EHR. Given the feed-forward architecture of our network, positional encodings plays a key role in filling the gap resulting from the lack of a recurrent structure that was historically the most common/successful approach for learning from sequences.

Age and visit segment are two embeddings that are unique to BEHRT to further empower it in dealing with the challenges we mentioned earlier. Age is known to be a key risk factor for most diseases (i.e., as we age, the risk of many diseases increase). By embedding age and linking it to each visit/diagnosis, not only do we provide the model with a sense of time (i.e., the time between events), but also we inform it of age as a universal epidemiological notion of when things happened (that is comparable across patients). Visit segment can be either A or B, which are two symbols to represent two trainable vectors in the segment embeddings; it changes alternatively between visits and it is used for providing BEHRT with extra information to indicate the separation between visits (i.e., visit segments for two adjacent visits of a patient will always be different).

Note that, there is no prescribed order for the multiple diagnoses within a visit. That is, for a given visit, the position, age, and segment embedding will be identical; this makes BEHRT order-invariant for intra-visit concepts (i.e., invariant to the ordering of diagnoses within a visit). Thus, attention mechanism purely investigates intra-visit relationships among diseases.

Through a unique combination of the four aforementioned embeddings, we provide the model with disease sequences, a precise sense of timing of events, and data about the delivery of care. In other words, the model has the ability to learn from the medical history and its corresponding age and visit patterns. All these, when combined, can paint a picture of one’s health that traditionally we might have sought to capture through many features manually extracted from EHR. Of course, we do not advocate for not using the full richness of the EHR and indeed see that as a follow up to this work. However, we aim to show how the complexity of our architecture when paired with this subset of EHR can still provide an accurate characterisation of one’s future health trajectory. BEHRT’s flexible architecture enables the use of additional concepts, e.g., by simply adding additional embeddings to the existing four.

### Pre-training BEHRT using masked language model (MLM)

In EHR data, just like language, it is intuitively reasonable to believe that a deep bidirectional model is more powerful than either a left-to-right model or the shallow concatenation of a left-to-right and a right-to-left model. Therefore, we pre-trained BEHRT using the same approach as the original BERT paper^18^, using MLM. We initialized disease, age, and segment embeddings randomly, and the positional encoding, as discussed previously, stems from a pre-determined encoding of position.When training the network and specifically, the embeddings for the MLM task, we left 86.5% of the disease words unchanged; 12% of the words were replaced with [mask]; and the remaining 1.5% of words, were replaced with randomly-chosen disease words.

Under this setting, BEHRT does not know which of the disease words are masked, so it stores a contextual representation of all of the disease words. Additionally, the small prevalence of change (i.e., only for 13.5% of all disease words) will not hamper the model’s ability to understand the EHR data. Lastly, the replacement of the disease words acts as injected noise into the model; it will distract the model from learning the true left and right context, and instead forces the model to fight through the noise and continue learning the overall disease trajectories. The pre-training MLM task was evaluated using precision score^34^, which calculates the ratio of true positive over the number of predicted positive samples (precision calculated at a threshold of 0.5). The average is calculated over all labels and over all patients. We see in Fig. 3b that the MLM classifier maps the tokens ${T}_{1}\ldots {T}_{N}$ to the masked words.

### Disease Prediction

In order to provide a comprehensive evaluation of BEHRT, we assess its learning in three predictive tasks: prediction of diseases in the next visit (T1), prediction of diseases in the next 6 months (T2), and prediction of diseases in the next 12 months (T3). In order to train our model and assess its predictions’ accuracy across these tasks, we first randomly allocated the patients into two groups: train and test (containing 80% and 20% of the patients, respectively). To define the training examples (i.e., input-output pairs) for T1, we randomly choose an index $j$ ($3 < j < {n}_{p}$) for each patient and form ${{\bf{x}}}_{p}=\{{{\bf{v}}}_{p}^{1},\ldots ,{{\bf{v}}}_{p}^{j}\}$ and ${{\bf{y}}}_{p}={{\bf{w}}}_{j+1}$, as input and output, respectively, where ${{\bf{w}}}_{j+1}$ is a multi-hot vector of length $G$, with 1’s, indexed for diseases that exist in ${{\bf{v}}}_{p}^{j+1}$. Note that each patient contributes *only one* input-output pair to the training and evaluation process.

For both T2 and T3, the formation of input and label are slightly modified. First, patients that do not have 6 or 12 months (for T2 and T3, respectively) worth of EHR (with or without a visit) after ${{\bf{v}}}_{p}^{4}$ will not be included in the analyses. Second, $j$ is chosen randomly from (3, n_*_), where n_*_ denotes the highest index after which there is 6 or 12 months (for T2 and T3, respectively) worth of EHR (with or without a visit). Lastly, ${{\bf{y}}}_{p}={{\bf{w}}}_{6m}$ and ${{\bf{y}}}_{p}={{\bf{w}}}_{12m}$ are multi-hot vectors of length $G$, with 1 for concepts/diseases that exist in the next 6 and 12 months, respectively. As a result of this final filtering of patients, we had 699 K, 391 K, and 342 K patients for T1, T2, and T3, respectively. A more comprehensive statistics for the population is shown in Supplementary Table S1. To clarify the task design, the BEHRT model is considered as “forced by design” to predict subsequent diseases in the patients’ medical history, and only patients who have at least one diagnosis in the coming visit/6 months/12 months are included.

We feed these input medical histories into BEHRT for feature extraction. Next, as shown in Fig. 3b, the network pools the information into a representation of the patient and passes it along to a single feed-forward classifier layer for output, subsequent visit prediction. Using this procedure, we train and test it three separate times for each of the three aforementioned tasks (T1–T3). We denote the model’s prediction for patient $p$ in the aforementioned tasks as ${{\bf{y}}}_{p}^{\ast }$, where the $i$th entry is the model’s prediction of that person having ${d}_{i}$. The evaluation metrics we used to compare **y**’s and y*’s, are area under the receiver operating characteristic curve (AUROC)^35^ and average precision score (APS)^36^; the latter is a weighted mean of precision and recall achieved at different thresholds. We calculated the APS and AUROC for each patient first, and then averaged the resulting APS and AUROC scores across all patients^35,36^.

To further investigate BEHRT’s predictive performance, we carried out three experiments: (1) We investigated if BEHRT can implicitly learn gender and utilise this latent understanding in subsequent visit prediction; (2) we carried out an ablation study by selectively deactivating age, segment, and/or position embeddings and seeing their effects on APS and AUROC; and (3) we assessed the model’s performance on the prediction of $new$ instances of diseases (i.e., predicting the labels/diagnoses, which had not appeared in one’s EHR history, at the time of prediction). The number of diseases considered is the same as that in T1, T2, and T3.

### Resources and Implementation

In this project, we have Python for coding our models and analyses pipelines. We relied on NVIDIA Titan Xp Graphical Processing Units (GPU) for pre-training, training, and testing. BertAdam^18^ is used as optimiser for both MLM and disease prediction tasks.

## Results

### Disease Embedding

We used Bayesian Optimisation^37^ to find the optimal hyperparameters for the MLM pre-training. The main hyperparameters here are the number of layers, the number of attention heads, hidden size, and “intermediate size” – see the original BERT paper for the details. This process resulted in an optimal architecture with 6 layers, 12 attention heads, intermediate layer size of 512, and hidden size of 288; For reproduceability purposes, we trained the MLM task for 100 epochs and the model’s performance was 0.6597 in precision score. Further details can be found in Supplementary Table S2.

**Figure 4:**
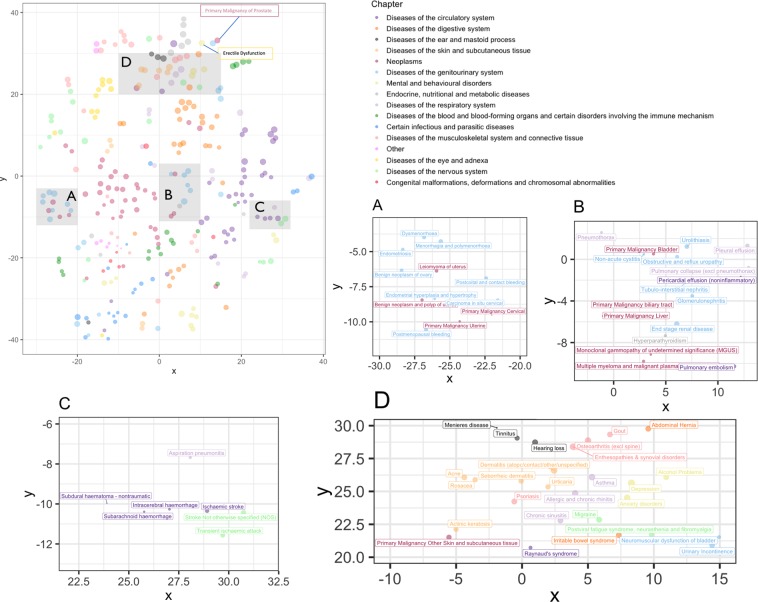
Visual investigation of the disease embedding. In this image we see a graph of disease embeddings projected in two dimensions where distance represents closeness of contextual association. The colors represent the Caliber chapter. Most associations are accepted by medical experts and maintain the gender-based divisions in illnesses, among other things. We zoom in and profile four clusters in this plot – shown in subfigures (**A**–**D**).

Given the importance of the disease embedding – resulting from training the MLM – and the effect that it can have on the overall prediction results, we first show the performance of our pre-training process, which mapped each one of the $G$ diseases to a 288-dimensional vector. Note that, for evaluating an embedding technique – even in NLP where the literature has a longer history and hence is more mature – there is not a single gold standard metric^38^. In this study, we devised three approaches: visual investigation (i.e., in comparison with medical knowledge), medical validation by clinical professional, and evaluation in a prediction task. For the former, we used t-SNE^39^ to reduce the dimensionality of the disease vectors to 2 – results are shown in Fig. 4. Based on the resulting patterns, we can see that diseases that are known to co-occur and/or belong to the same clinical groups, are grouped together. Note that, while most neighborhoods make sense and are aligned with medical knowledge, there are diseases clusters that (due to extreme dimensionality reduction, for instance) might seem counter-intuitive.

A reassuring pattern that can be seen in Fig. 4, for instance, is the natural stratification of gender-specific diseases. For instance, diseases that are unique to women (e.g., endometriosis, dysmenorrhea, menorrhagia, …) are quite distant from those that are unique to men (e.g., erectile dysfunction, primary malignancy of prostate, …). Such patterns seem to suggest that our disease embedding built an understanding of the context in which diagnoses happen, and hence infer factors such as gender that it is not explicitly fed.

Furthermore, the colour in Fig. 4 represent the original Caliber disease chapters (see the legends in the main subplot). As can be seen, natural clusters are formed that in most cases consist of disease of the same chapter (i.e., the same colour). Some of these clusters, however, are correlated but not identical to these chapters; for instance, many eye and adnexa diseases are amongst nervous system diseases and many nervous system disease are also among many musculoskeletal diseases. Overall, this map can be seen as diseases’ correspondence to each other based on 1.6 million people’s EHR.

Lastly, for each disease that occurred in at least 1% of the population (i.e., 87 diseases), we found the ten closest diseases (using cosine similarity of their embeddings). Comparing these top-10 neighbourhoods against those provided by a clinical researcher, we found a 0.757 overlap (i.e., nearly 76% of our 87 top-10 neighbourhoods were seen as clinically valid by a clinical expert). A full table of results for the 87 diseases is offered in Supplementary Table S3. The clinical researcher notes that while many of the most similar associations had clear overlap in symptomatology, some were graded to be poor disease associations. Thus, the researcher concludes that BEHRT has a strong ability to understand the latent characteristics of the disease, without them being explicitly given to it.

### Attention and Interpretability

**Figure 5:**
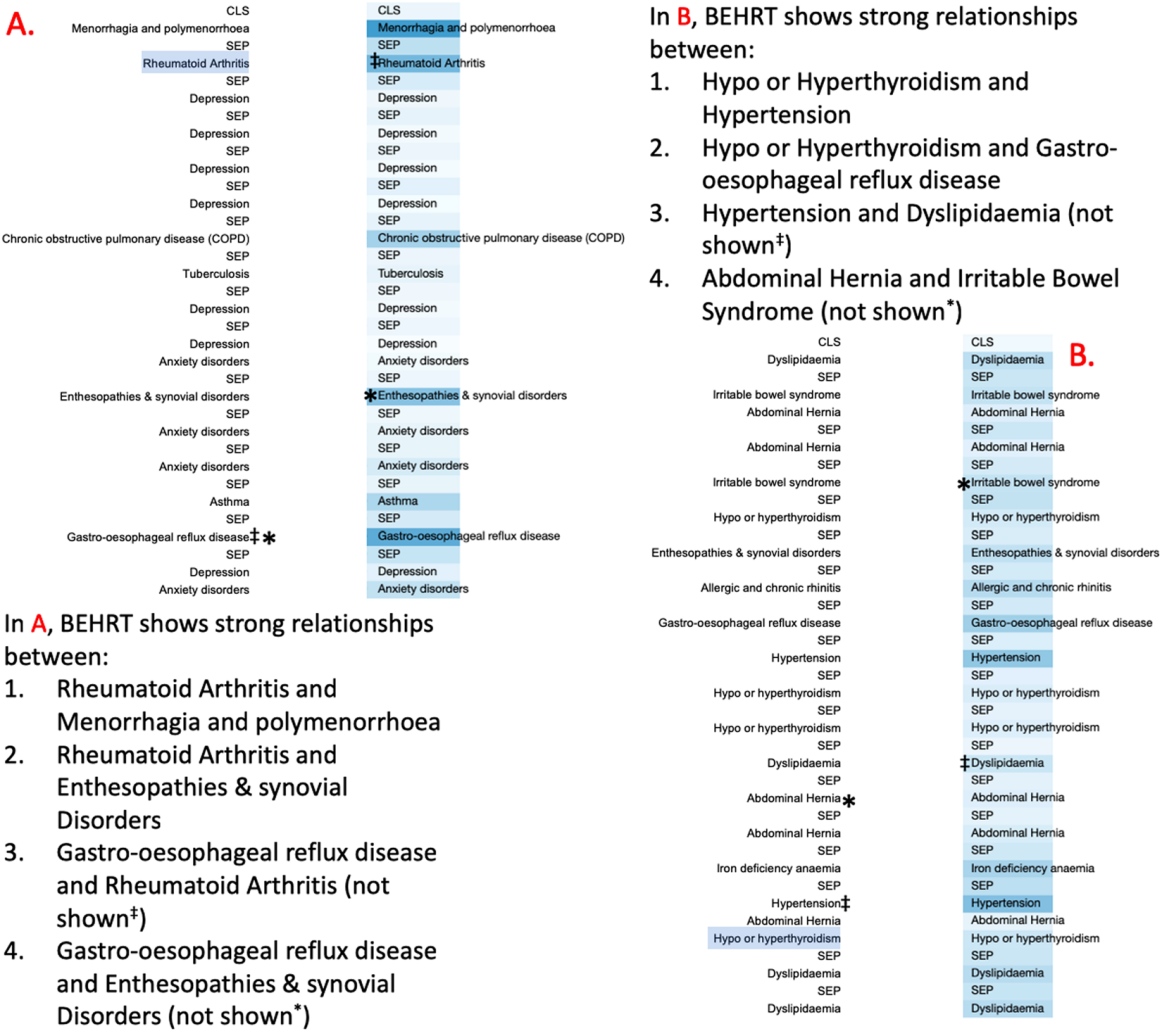
The analysis of BEHRT’s self-attention. This figure shows the EHR history of patients (**A** and **B**), each presented as two identical columns (shown chronologically, going downwards) for the convenience of association analysis. The left side of the column represents the disease of interest and the right column indicates the corresponding associations to the highlighted disease on the left. The intensity of the blue on the right column represents the strength of the attention score – the stronger the intensity, the stronger the association and hence the stronger the attention score. The attention scores are specifically retrieved from the attention component of the last layer of BEHRT network.

Another interesting property of BEHRT is its self-attention mechanism; this gives it the ability to find the relationships among events which goes beyond temporal/sequence adjacency. This self-attention mechanism is able to unearth deeper and more complex relationships between a diagnosis in one visit and other diagnoses. We analyse the attention-based patterns for patients using the approach introduced by Vig^40^. These results for two example patients are shown in Fig. 5. Note that, since BEHRT is bidrectional, the self-attention mechanism captures non-directional relationships among diseases (i.e., their correspondence with each other, rather than one causing the other).

For patient A in Fig. 5, for example, the self-attention mechanism has shown strong connections between rheumatoid arthritis and enthesopathies and synovial disorders (far in the future of the patient). This is a great example of where attention can go beyond recent events and find long-range dependencies among diseases. Note that, as described earlier and illustrated in Fig. 3, the sequence we model is a combination of four embedding (disease, age, segment, and position) that go through layers of transformations to form a latent space abstraction. While in Fig. 5 we labelled the cells with disease names, a more precise labelling will be diseases in their context (e.g., at a given age and visit).

### Disease Prediction

BEHRT after the MLM pre-training, can be considered a universal EHR feature extractor that only with a small additional training can be employed for a range of downstream tasks. In this work, the downstream task of choice is the multi-disease prediction problem that we described earlier. The results from the evaluation of the model’s performance is shown in Table 1. This table demonstrates BEHRT’s superior predictive power compared to two of the most successful approaches in the literature (i.e., Deepr^12^ and RETAIN^17^). To demonstrate a fair comparison of models, we carefully analyse model architecture for Deepr and RETAIN. We note Deepr models time between visits using special “words” and utilises demographic information (gender) for prediction. Regarding RETAIN, in addition to including gender to bolster predictive power, we implement an architecture endorsed by the author^41^ that would augment the original architecture’s performance: we encode time for the visits, as well as bidirectionality for the RNN framework. We used Bayesian Optimisation to find the optimal hyperparameters for RETAIN and Deepr before the evaluation. More details on the hyperparameter search and optimisation can be found in Supplementary Tables S4 and S5. The three supervised subsequent prediction task models were trained for 15–20 epochs.

**Table 1:** Model performances in the prediction tasks.

Model Name	Next Visit (APS|AUROC)	Next 6 M (APS|AUROC)	Next 12 M (APS|AUROC)
**BEHRT**	**0.462**|**0.954**	**0.525**|**0.958**	**0.506**|**0.955**
Deepr	0.360|0.942	0.393|0.943	0.393|0.943
RETAIN	0.382|0.921	0.417|0.927	0.413|0.928

**Figure 6:**
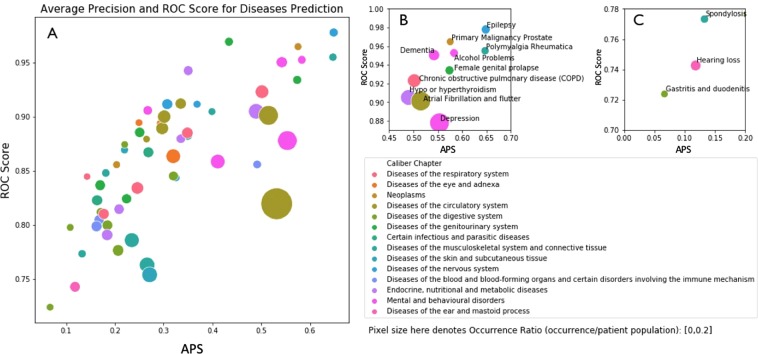
Disease-wise precision analysis. Each circle in these graphs represents a disease, and its colour and size denote the Caliber chapter and prevalence, respectively. Also, in these plots, we show APS and AUROC on the x- and y-axis, respectively. Therefore, the further right and higher a disease, the better BEHRT’s job at predicting its occurrence in the next 6 months. Subplot (**A**) illustrated the full results, and subplots (**B** and **C**) illustrate the best and worst sections of the plot, in terms of BEHRT’s performance.

Besides comparing the APS, which provides an average view across all patients and all thresholds, we also assessed the model’s performance for predicting for each disease. To do so for a given disease ${d}_{i}$, we only considered the $i$th entry in ${{\bf{y}}}_{p}$ and ${{\bf{y}}}_{p}^{\ast }$ vectors and calculated AUROC and APS scores, as well as their respective occurrence ratios (percent of patients that have a given disease in their EHR) for comparison. The results for T2 (or, next 6-months prediction task) is shown in Fig. 6. For visual convenience and in our analysis, we did not include rare diseases with prevalence of less than 1% in our data.

The result shows that BEHRT is able to make predictions with relatively high precision and recall for diseases such as epilepsy (0.016), primary malignancy prostate (0.011), polymyalgia rheumatica (0.013), hypo or hyperthyroidism (0.047), and depression (0.0768). A numerical summary of this analysis can be found in Supplementary Table S6. Furthermore, a comparison of the general APS/AUROC trends across the three models can be found in Supplementary Fig. S1.

To investigate the model’s performance further, we carried out an analysis to assess the model’s ability to learn gender-related concepts, without being given gender as an input variable; this “gender analysis” was done on the results of the T2 task. Looking at the gender-specific diseases (e.g., endometriosis, female genital prolapse, and menorrhagia and polymenorrhoea that are unique to women, and hyperplasia of prostate, male infertility and erectile dysfunction that are unique to men), Supplementary Table S7 shows that, except for a few predictions, BEHRT has correctly avoided matching male diseases to female patients and vice versa. One of the seemingly gender errors in our results, is “male infertility”, which has been assigned to female patients; after further investigations, we discovered that “male infertility” is recorded for both male (365) and female (1,734) patients; we also saw records of “female infertility” for both male (192) and female (2,306) patients. This was due to gender-agnostic Read codes (K26y300, K26y400, K5B1100, K5By000, and others) mapped to both “male infertility” and “female infertility” in the Caliber system. Disregarding “male infertility” and “female infertility”, we see BEHRT naturally identifies gender in feature extraction and can make robust gender-specific conclusions for subsequent disease prediction.

As mentioned earlier, BEHRT can accommodate variety of input concepts that exist in EHR. Therefore, selection of the appropriate concepts can be an important aspect of designing the BEHRT architecture. Therefore, we carried out an ablation study, where we selectively deactivated parts of the input space and assessed the model’s performance for each resulting scenario; see Supplementary Table S8. While the results are not changing significantly in terms of AUROC, we see a much bigger range in APS. Overall, the results illustrate that position and age are important features in modelling the EHR sequences; they both improved the baseline (i.e., disease-only) model’s performance significantly. In contrast, adding segment to the baseline model resulted in a very small (almost negligible) improvement.

Lastly, for a subset of diseases in the label that are occurring for the first time (first incidence), we calculate predictive performance of the three models in Table 2. BEHRT shows superior predictive performance in all three tasks with respect to RETAIN and Deepr. The ranking of the three models in terms of performance (both AUROC/APS) is identical to the ranking shown in Table 1.

**Table 2:** Model performances in the prediction tasks - First Incidence of Diseases.

Model Name	Next Visit (APS|AUROC)	Next 6 M (APS|AUROC)	Next 12 M (APS|AUROC)
**BEHRT**	**0.216**|**0.904**	**0.228**|**0.907**	**0.226**|**0.905**
Deepr	0.095|0.800	0.104|0.814	0.098|0.805
RETAIN	0.108|0.836	0.115|0.845	0.109|0.836

## Discussion

In this paper, we introduced a novel deep neural network model for EHR called BEHRT; an interpretable personalised risk model, which scales across a range of diseases and incorporates a wide range of EHR modalities/concepts in its modular architecture. BEHRT can be pre-trained on a large dataset and then with small fine tuning will result in a striking performance in a wide range of downstream tasks. We demonstrated this property of the model by training and testing it on CPRD - one of the largest linked primary care EHR systems – for predicting the next mostly likely diseases in one’s future visits. Based on our results, BEHRT outperformed the best deep EHR models in the literature by more than ~8% (absolute improvement) in predicting for a range of more than 300 diseases.

BEHRT offers a flexible architecture that is capable of capturing more modalities of EHR data than we have already demonstrated in this paper. We demonstrated this flexibility of BEHRT’s modular architecture by designing a model that employed 4 key concepts from the EHR: diseases, age, segment, and position. Through this mix, the model will not only have the ability to learn about the past diseases and their relationships with future diagnoses (i.e., learning disease trajectories), but also gain insights about the underlying generating process of EHR; we can refer to this as the practice of care. In other words, the model will learn distributed/complex representations that are capable of capturing concepts such as “this patient had diseases A and B at a young age, and suddenly, the frequency of visits increased, and a new diagnosis C appeared; all these, plus the patient’s age, will increase the chance of disease D happening next”. In future works, one can add more to these four concepts and bring medication, tests, and interventions to the model with minimum architectural changes – by only adding new rows to the architecture depicted in Fig. 3a.

Our primary objective in this study was to provide the field with an accurate model for the prediction of next/future diseases. While doing this, BEHRT provides multiple byproducts that each can be useful on their own and/or as a key component of future works. For instance, the disease embeddings resulting from BEHRT can provide great insights into how various diseases are related to each other; it goes beyond simple disease co-occurrence and rather learns the closeness of diseases based on their trajectories in a big population of patients. Furthermore, such pre-trained disease embeddings can be used by future researchers as a reliable disease vectors, ready for numeric/algebraic operations. This is very similar to fields such as NLP, where sharing word vectors for other researchers is a common practice. Additionally, we have shown that the disease correspondences that result from BEHRT’s attention mechanism can be useful for illustrating the disease trajectories for multi-morbid patients; not only it shows how diseases co-occur, but also it shows the influence of certain diseases in one’s past on their future risk of other diseases. These correspondences are not strictly temporal but rather contextual. Through our analyses of the supervised prediction tasks performance (T1, T2, and T3), we first see that BEHRT has the ability to make robust, gender-specific predictions without inclusion of gender. We also note that the ablation study has showed us important embeddings for inclusion; while position was important for this task, we note that perhaps in diagnosing age-related diseases (e.g. Alzheimer’s disease, arthritis, etc), the age embedding might be more vital for predictive power. Thirdly, we conclude that even for incident disease prediction, BEHRT performs better than RETAIN and Deepr. As a future work, we aim to provide these attention-visualisation tools to medical researchers to help them better understand the contextual meaning of a diagnosis in the midst of other diagnoses of patients. Through this tool, medical researchers can even craft medical history timelines based on certain diseases or patterns and in a way, query our BEHRT model and visualiser to perhaps uncover novel disease contexts.

Despite exploring some of the properties of BEHRT, there are a range of future works that one can base on BEHRT’s architecture and properties. For instance, BEHRT’s ability to summarise a patient’s health journey into a simple vector enables its use in a wide range of machine learning and exploratory analysis frameworks. That is, in most such analyses, one requires to measure the similarities between two medical concepts and/or two patients; this has been shown to be achievable, regardless of the variabilities that are inherent in EHR data, going from one patient to the other. Another direction of future research, can be the use of BEHRT for other downstream tasks such as single-disease prediction tasks, or non-diagnosis tasks such as prediction of hospital readmission and/or mortality – all these are extremely important clinical events. In such analyses, BEHRT’s interpretability can also be a useful feature to understand various pathways that will lead to a disease/event or result from a disease/event. To improve BEHRT’s accuracy, also, one can rely on some of the well-known frameworks such as ensembles of BEHRTs that have been shown to be effective for other learners.

## Supplementary information

Supplementary Information.

## Data Availability

The data that support the findings of this study are available from Clinical Practice Research Datalink (CPRD). The link: https://www.cprd.com/Data explains more in depth about the nature and accessibility of the data. Furthermore, regarding accessibility, https://www.cprd.com/primary-care explains: “Access to data from CPRD is subject to a full licence agreement containing detailed terms and conditions of use. Patient level datasets can be extracted for researchers against specific study specifications, following protocol approval from the Independent Scientific Advisory Committee (ISAC).” Thus, restrictions apply to the availability of these data, which were used under license for the current study, and so are not publicly available.
