# Supplementary material for: BEHRT: Transformer for Electronic Health Records
Source: Sci Rep. 2020 Apr 28;10:7155. doi: 10.1038/s41598-020-62922-y (PMC7189231; doi:10.1038/s41598-020-62922-y)
Supplement: Supplementary file 1 — Supplementary Information. [file 41598_2020_62922_MOESM1_ESM.pdf]

BEHRT: Transformer for Electronic Health Records

Mr. Yikuan Li , Mr. Shishir Rao, Dr. Jose Roberto Ayala Solares, Dr. Abdelaali Hassaine, Dr. Rema Ramakrishnan, Dr. Dexter Canoy, Dr. Yajie Zhu, Dr. Kazem Rahimi, Dr. Gholamreza Salimi-Khorshidi

Supplementary Information

Legend:
Table S1:This table provides the basic statistics for the characteristics of the selected cohorts.

Table S2: This table shows the Hyperparameter tuning results for BEHRT. The columns are various hyperparameters and the rows are different runs. The bold items are best performing.

Table S3: This table shows the top ten associations for each disease that occurred in at least 1% of the population. Each row is a disease and the columns are the ten diseases associated to said disease. The annotation "Good" means that the medical validator defined the association as a medically valid association and "Not Good" means that the medical validator defined the association as medically invalid.

Table S4: This table shows the Hyperparameter tuning results for Deepr. The columns are various hyperparameters and the rows are different runs. The bold items are best performing.

Table S5: This table shows the Hyperparameter tuning results for RETAIN. The columns are various hyperparameters and the rows are different runs. The bold items are best performing.

Table S6: This table shows the individual, disease wise precision for BEHRT for the next 6 months prediction task. The ratio in column 5 means the occurrence of a particular disease code in the label of the task over all labels in test data.

Figure S1: This figure shows the graph for an understanding of APS and AUROC for the next 6 months prediction task for all three models denoted by colour of dot.

Table S7: This table shows the number of Male patient Predictions and Female patient Predictions with predictive probability above 0.5 for a gender-specific disease in the next 6 months prediction test dataset. Disease Gender represents whether a disease is a male or female specific disease and M and F implies Male and Female respectively.

Table S8: This table shows the performance of the ablation study conducted on the next 6 Months prediction task. By turning on and off various embeddings, we measure performance with Average Precision Score (APS) and Area Under the ROC Curve (AUROC).

Population Statistics

Table S1: Statistics of Cohorts Selected Prediction Tasks

Characteristics		Next Visit	Next 6 M	Next 12M
Gender	Male	41.80%	42.30%	41.70%
	Female	58.20%	57.70%	58.30%
Ethnicity	White	46.40%	48.30%	47.40%
	Unknown	43.80%	44.00%	44.50%
	Indian	0.40%	0.50%	0.50%
	Other	0.30%	0.30%	0.30%
	Pakistani	0.20%	0.30%	0.20%
	Black Carib	0.20%	0.30%	0.20%
	Other Asian	0.10%	0.10%	0.10%
	Black African	0.10%	0.10%	0.10%
	Mixed	0.10%	0.10%	0.10%
	Bangladeshi	0.08%	0.07%	0.07%
	Black Other	0.07%	0.06%	0.06%
	Chinese	0.06%	0.06%	0.05%
Age Start	0.25 Quantile	45	46	46
	0.5 Quantile	58	60	59
	0.75 Quantile	70	71	70
Age End	0.25 Quantile	56	58	58
	0.5 Quantile	70	71	71
	0.75 Quantile	81	82	82
Unique Codes	0.25 Quantile	7	8	8
	0.5 Quantile	9	10	11
	0.75 Quantile	12	15	15
Number of Visits	0.25 Quantile	10	14	14
	0.5 Quantile	15	20	20
	0.75 Quantile	24	30	30
Age Start and Age End: Corresponding age for the first visit and last visit been recorded				

Hyperparameter Tuning for MLM

In this section, we show the hyperparameter tuning results. In Table S2, we show the model's performance during the process of training the MLM. We performed Bayesian Optimisation to search for optimal parameters for the model.

Table S2. MLM Hyperparameter Tuning

Iteration	Hidden Size	Layers	Attention Heads	Intermediate Size	Precision
1	216	3	6	256	0.6191
2	288	9	12	512	0.6399
3	216	3	12	512	0.6175
4	432	3	18	512	0.6397
5	288	6	6	784	0.6380
6	216	6	18	512	0.6262
7	288	3	18	512	0.6292
8	432	3	6	784	0.6426
9	288	6	12	512	0.6356
10	288	3	12	256	0.6283
11	432	9	18	512	0.6466
12	576	9	6	1024	0.6538
13	432	3	18	1024	0.6411
14	432	9	6	1024	0.6508
15	576	6	6	256	0.6503
16	576	6	12	256	0.6510
17	360	9	18	512	0.6404
18	576	9	6	512	0.6513
19	288	6	6	512	0.6363
20	288	3	6	512	0.6297
**21**	**288**	**6**	**12**	**512**	**0.6597**
22	576	3	12	512	0.6487
23	360	6	6	784	0.6412
24	432	9	6	512	0.6497
25	360	6	12	512	0.6423

Medical Evaluation of Associations

Table S3 shows the medical evalution of our diseases associations based on highest consine similarity. Each row is a disease and the columns are the ten diseases associated to said disease. The annotation "Good" means that the medical validator defined the association as a medically valid association and "Not Good" means that the medical validator defined the association as medically invalid.

Table S3: Medical Validation of Disease Associations based on Disease Embeddings

	Disease	1	2	3	4	5	6	7	8	9	10
1	Postcoital and contact bleeding	Postcoital and contact bleeding-Good	Carcinoma in situ cervical-Good	Dysmenorrhoea-Not Good	Leiomyoma of uterus-Good	Endometriosis-Good	Primary Malignancy Cervical-Good	Menorrhagia and polymenorrhoea-Not Good	Prematurity-Not Good	Polycystic ovarian syndrome-Good	Endometrial hyperplasia and hypertrophy-Good
2	Cholecystitis	Cholecystitis-Good	Cholelithiasis-Good	Cholangitis-Good	Pancreatitis-Good	Appendicitis-Not Good	Diaphragmatic hernia-Not Good	Infection of liver-Good	Fatty Liver-Good	Benign neoplasm of stomach and duodenum-Not Good	Supraventricular tachycardia-Not Good
3	Acute Kidney Injury	Acute Kidney Injury-Good	Right bundle branch block-Good	Pulmonary collapse (excl pneumothorax)-Good	Urinary Tract Infections-Good	Atrioventricular block, first degree-Good	Atrioventricular block, complete-Good	Lower Respiratory Tract Infections-Good	Pericardial effusion (noninflammatory)-Good	Trifascicular block-Good	Secondary Malignancy retroperitoneum and peritoneum-Good
4	Benign neoplasm of ovary	Benign neoplasm of ovary-Good	Endometriosis-Good	Benign neoplasm and polyp of uterus-Good	Endometrial hyperplasia and hypertrophy-Good	Leiomyoma of uterus-Good	Dysmenorrhoea-Good	Primary Malignancy Uterine-Good	Postmenopausal bleeding-Good	Primary Malignancy Cervical-Good	Polycystic ovarian syndrome-Good

5	Visual impairment and blindness	Visual impairment and blindness-Good	Macular degeneration-Good	Retinal vascular occlusions-Good	Retinal detachment-Good	Keratitis-Good	Posterior Uveitis-Good	Glaucoma-Good	Delirium, not induced by alcohol and other psychoactive substances-Not Good	Anterior and Intermediate Uveitis-Good	Cataract-Good
6	Intervertebral disc disorders	Intervertebral disc disorders-Good	Spondylolisthesis-Good	Spinal stenosis-Good	Spondylosis-Good	Scoliosis-Not Good	Peripheral neuropathies (excluding cranial nerve and carpal tunnel syndromes)-Good	Postinfective and reactive arthropathies-Not Good	Alopecia areata-Not Good	Infection of bones and joints-Not Good	Disorders of autonomic nervous system-Good
7	Peptic ulcer disease	Peptic ulcer disease-Good	Gastritis and duodenitis-Good	Diaphragmatic hernia-Not Good	Peritonitis-Good	Benign neoplasm of stomach and duodenum-Good	Oesophagitis and oesophageal ulcer-Good	Appendicitis-Good	Barrett's oesophagus-Good	Infections of the digestive system-Good	Cholelithiasis-Not Good
8	Fibromatoses	Fibromatoses-Good	Retinal detachment-Good	Primary Malignancy Malignant Melanoma-Good	Benign essential tremor-Not Good	Disorders of autonomic nervous system-Not Good	Hyposplenism-Good	Primary Malignancy Other Skin and subcutaneous tissue-Good	Primary or Idiopathic Thrombocytopaenia-Not Good	Nasal polyp-Good	Subdural haematoma - nontraumatic-Not Good
9	Fracture of wrist	Fracture of wrist-Good	Osteoporosis-Good	Fracture of hip-Good	Scoliosis-Not Good	Collapsed vertebra-Good	Fibromatoses-Good	Carpal tunnel syndrome-Good	Primary pulmonary hypertension-Not Good	Menieres disease-Good	Delirium, not induced by alcohol and other psychoactive substances-Good

10	Ischaemic stroke	Ischaemic stroke-Good	Intracerebral haemorrhage-Good	Stroke Not otherwise specified (NOS)-Good	Transient ischaemic attack-Good	Aspiration pneumonitis-Good	Subarachnoid haemorrhage-Good	Subdural haematoma - nontraumatic-Good	Delirium, not induced by alcohol and other psychoactive substances-Good	Bell's palsy-Not Good	Syndrome of inappropriate secretion of antidiuretic hormone-Good
11	Primary Malignancy Breast	Primary Malignancy Breast-Good	Primary Malignancy Uterine-Good	Postmenopausal bleeding-Good	Primary Malignancy Cervical-Good	Secondary Malignancy Other organs-Good	Secondary Malignancy Lymph Nodes-Good	Benign neoplasm and polyp of uterus-Not Good	Primary Malignancy Lung and trachea-Not Good	Primary Malignancy Malignant Melanoma-Not Good	Primary Malignancy Thyroid-Not Good
12	Pleural effusion	Pleural effusion-Good	Pericardial effusion (noninflammatory)-Good	Pulmonary collapse (excl pneumothorax)-Good	Secondary Malignancy Pleura-Good	Infections of Other or unspecified organs-Good	Secondary pulmonary hypertension-Good	Primary pulmonary hypertension-Good	Atrial Fibrillation and flutter-Not Good	Primary Malignancy Other Organs-Good	Pleural plaque-Good
13	Peripheral neuropathies (excluding cranial nerve and carpal tunnel syndromes)	Peripheral neuropathies (excluding cranial nerve and carpal tunnel syndromes)-Good	Diabetic neurological complications-Good	Disorders of autonomic nervous system-Good	Intervertebral disc disorders-Good	Carpal tunnel syndrome-Not Good	Spinal stenosis-Good	Postviral fatigue syndrome, neurasthenia and fibromyalgia-Good	Erectile dysfunction-Good	Peripheral arterial disease-Good	Spondylolisthesis-Good
14	Anal fissure	Anal fissure-Good	Anorectal fistula-Not Good	Pilonidal cyst-Not Good	Irritable bowel syndrome-Good	Benign neoplasm of colon, rectum, anus and anal canal-Not Good	Ulcerative colitis-Good	Haemangioma (any site)-Not Good	Alopecia areata-Not Good	Postcoital and contact bleeding-Not Good	Vitiligo-Good

15	Postmenopausal bleeding	Postmenopausal bleeding-Good	Benign neoplasm and polyp of uterus-Good	Primary Malignancy Uterine-Good	Endometrial hyperplasia and hypertrophy-Good	Leiomyoma of uterus-Good	Benign neoplasm of ovary-Not Good	Primary Malignancy Breast-Good	Female genital prolapse-Good	Primary Malignancy Cervical-Good	Postcoital and contact bleeding-Good
16	Venous thromboembolic disease (Excl PE)	Venous thromboembolic disease (Excl PE)-Good	Pulmonary embolism-Good	Bell's palsy-Not Good	Pleural effusion-Good	Primary Malignancy Other Organs-Good	Fracture of hip-Good	Peritonitis-Good	Cholangitis-Good	Thrombophilia-Good	Atrial Fibrillation and flutter-Good
17	Fracture of hip	Fracture of hip-Good	Collapsed vertebra-Good	Osteoporosis-Good	Fracture of wrist-Good	Subdural haematoma - nontraumatic-Not Good	Delirium, not induced by alcohol and other psychoactive substances-Good	Dementia-Good	Hyperparathyroidism-Good	Right bundle branch block-Not Good	Essential tremor-Not Good
18	Macular degeneration	Macular degeneration-Good	Visual impairment and blindness-Good	Cataract-Good	Retinal detachment-Good	Retinal vascular occlusions-Good	Glaucoma-Good	Diabetic ophthalmic complications-Good	Anterior and Intermediate Uveitis-Good	Eye infections-Not Good	Diabetic neurological complications-Good
19	Benign neoplasm of colon, rectum, anus and anal canal	Benign neoplasm of colon, rectum, anus and anal canal-Good	Diverticular disease of intestine (acute and chronic)-Good	Benign neoplasm of stomach and duodenum-Good	Angiodysplasia of colon-Good	Primary Malignancy colorectal and anus-Good	Anorectal fistula-Good	Anal fissure-Good	Diaphragmatic hernia-Not Good	Barrett's oesophagus-Good	Peritonitis-Good
20	Postviral fatigue syndrome, neurasthenia and fibromyalgia	Postviral fatigue syndrome, neurasthenia and fibromyalgia-Good	Sjogren's disease-Good	Lupus erythematosus (local and systemic)-Good	Migraine-Not Good	Irritable bowel syndrome-Good	Peripheral neuropathies (excluding cranial nerve and carpal tunnel syndromes)-Good	Intervertebral disc disorders-Good	Spondylolisthesis-Good	Neuromuscular dysfunction of bladder-Good	Leiomyoma of uterus-Good

21	Dysmenorrhoea	Dysmenorrhoea-Good	Endometriosis-Good	Leiomyoma of uterus-Good	Menorrhagia and polymenorrhoea-Not Good	Postcoital and contact bleeding-Not Good	Carcinoma in situ cervical-Not Good	Endometrial hyperplasia and hypertrophy-Good	Polycystic ovarian syndrome-Good	Prematurity-Not Good	Female pelvic inflammatory disease-Good
22	Cholelithiasis	Cholelithiasis-Good	Cholecystitis-Good	Cholangitis-Good	Pancreatitis-Good	Gastritis and duodenitis-Not Good	Scleritis and episcleritis-Not Good	Appendicitis-Not Good	Diaphragmatic hernia-Not Good	Peptic ulcer disease-Not Good	Obesity-Good
23	Primary Malignancy Prostate	Primary Malignancy Prostate-Good	Secondary Malignancy Bone-Good	Primary Malignancy Bladder-Good	Hyperplasia of prostate-Good	Secondary Malignancy Other organs-Good	Obstructive and reflux uropathy-Not Good	Erectile dysfunction-Good	Asbestosis-Good	Secondary malignancy Liver and intrahepatic bile duct-Good	Hyposplenism-Good
24	Myocardial Infarction	Myocardial Infarction-Good	Unstable Angina-Good	Stable Angina-Good	Coronary heart disease not otherwise specified-Good	Left bundle branch block-Good	Heart failure-Good	Atrioventricular block, second degree-Good	Ventricular tachycardia-Good	Atrioventricular block, first degree-Good	Atrioventricular block, complete-Good
25	Peripheral arterial disease	Peripheral arterial disease-Good	Abdominal Aortic Aneurysm-Good	Diabetic neurological complications-Good	Unstable Angina-Good	Retinal vascular occlusions-Good	Transient ischaemic attack-Good	Stable Angina-Good	Type 1 Diabetes Mellitus, Type 2 Diabetes Mellitus, and Diabetes Mellitus ‚Äì other or not specified-Good	Visual impairment and blindness-Good	Coronary heart disease not otherwise specified-Good
26	Diaphragmatic hernia	Diaphragmatic hernia-Good	Gastritis and duodenitis-Good	Benign neoplasm of stomach and duodenum-Not Good	Gastro-oesophageal reflux disease-Good	Barrett's oesophagus-Good	Peptic ulcer disease-Good	Oesophagitis and oesophageal ulcer-Good	Diverticular disease of intestine (acute and chronic)-Not Good	Benign neoplasm of colon, rectum, anus and anal canal-Not Good	Cholelithiasis-Not Good

27	Urolithiasis	Urolithiasis-Good	Obstructive and reflux uropathy-Good	Tubulo-interstitial nephritis-Not Good	Hyperplasia of prostate-Good	Appendicitis-Not Good	Non-acute cystitis-Good	Primary Malignancy Bladder-Good	Pancreatitis-Good	Bell's palsy-Not Good	Cholecystitis-Not Good
28	Glaucoma	Glaucoma-Good	Retinal vascular occlusions-Good	Visual impairment and blindness-Good	Macular degeneration-Good	Cataract-Good	Anterior and Intermediate Uveitis-Good	Keratitis-Good	Diabetic ophthalmic complications-Good	Parkinson's disease-Good	End stage renal disease-Good
29	Urinary Tract Infections	Urinary Tract Infections-Good	Ear and Upper Respiratory Tract Infections-Not Good	Infections of the digestive system-Good	Female pelvic inflammatory disease-Good	Lower Respiratory Tract Infections-Good	Infection of other or unspecified genitourinary system-Good	Infection of male genital system-Good	Acute Kidney Injury-Good	Infection of skin and subcutaneous tissues-Not Good	Infection of bones and joints-Good
30	Tinnitus	Tinnitus-Good	Hearing loss-Good	Menieres disease-Good	Raynaud's syndrome-Good	Haemangioma (any site)-Good	Seborrheic dermatitis-Not Good	Primary Malignancy Malignant Melanoma-Good	Irritable bowel syndrome-Not Good	Chronic sinusitis-Good	Peripheral neuropathies (excluding cranial nerve and carpal tunnel syndromes)-Not Good
31	Lower Respiratory Tract Infections	Lower Respiratory Tract Infections-Good	Infections of the Heart-Not Good	Infection of liver-Good	Infection of other or unspecified genitourinary system-Not Good	Urinary Tract Infections-Not Good	Ear and Upper Respiratory Tract Infections-Good	Infections of the digestive system-Not Good	Infection of bones and joints-Not Good	Infection of male genital system-Not Good	Agranulocytosis-Good
32	Transient ischaemic attack	Transient ischaemic attack-Good	Ischaemic stroke-Good	Stroke Not otherwise specified (NOS)-Good	Intracerebral haemorrhage-Good	Retinal vascular occlusions-Good	Subarachnoid haemorrhage-Not Good	Bell's palsy-Not Good	Peripheral arterial disease-Good	Delirium, not induced by alcohol and other psychoactive substances-Good	Nonrheumatic aortic valve disorders-Good

33	Bacterial Diseases (excl TB)	Bacterial Diseases (excl TB)-Good	Other or unspecified infectious organisms-Good	Viral diseases (excl chronic hepatitis/HIV)-Not Good	Parasitic infections-Not Good	Tubulo-interstitial nephritis-Good	Female infertility-Good	Obstructive and reflux uropathy-Good	Acute Kidney Injury-Good	Aspiration pneumonitis-Good	Infections of Other or unspecified organs-Good
34	Polymyalgia Rheumatica	Polymyalgia Rheumatica-Good	Giant Cell arteritis-Good	Monoclonal gammopathy of undetermined significance (MGUS)-Not Good	Rheumatoid Arthritis-Not Good	Collapsed vertebra-Good	Spinal stenosis-Good	Epilepsy-Good	Spondylolisthesis-Good	Menieres disease-Good	Nonrheumatic aortic valve disorders-Good
35	Primary Malignancy Other Skin and subcutaneous tissue	Primary Malignancy Other Skin and subcutaneous tissue-Good	Actinic keratosis-Good	Primary Malignancy Malignant Melanoma-Good	Haemangioma (any site)-Not Good	Primary Malignancy Oropharyngeal-Not Good	Fibromatoses-Good	Rosacea-Not Good	Seborrheic dermatitis-Not Good	Hyperplasia of prostate-Not Good	Gout-Good
36	Urticaria	Urticaria-Good	Dermatitis (atopc/contact/other/unspecified)-Good	Seborrheic dermatitis-Not Good	Keratitis-Good	Chronic sinusitis-Good	Lichen planus-Not Good	Hidradenitis suppurativa-Not Good	Benign neoplasm of ovary-Not Good	Alopecia areata-Good	Raynaud's syndrome-Not Good
37	Actinic keratosis	Actinic keratosis-Good	Primary Malignancy Other Skin and subcutaneous tissue-Good	Primary Malignancy Malignant Melanoma-Good	Haemangioma (any site)-Not Good	Hearing loss-Not Good	Osteoarthritis (excl spine)-Good	Fibromatoses-Not Good	Seborrheic dermatitis-Good	Rosacea-Good	Enthesopathies & synovial disorders-Not Good
38	Rosacea	Rosacea-Good	Acne-Not Good	Seborrheic dermatitis-Good	Primary Malignancy Other Skin and subcutaneous tissue-Good	Ulcerative colitis-Good	Migraine-Good	Actinic keratosis-Good	Vitiligo-Not Good	Urticaria-Not Good	Female genital prolapse-Not Good

39	Osteoporosis	Osteoporosis-Good	Collapsed vertebra-Good	Fracture of wrist-Good	Fracture of hip-Good	Scoliosis-Good	Primary Malignancy Breast-Good	Spondylosis-Good	Cataract-Good	Osteoarthritis (excl spine)-Not Good	Hyperparathyroidism-Good
40	Gastritis and duodenitis	Gastritis and duodenitis-Good	Diaphragmatic hernia-Not Good	Peptic ulcer disease-Good	Gastro-oesophageal reflux disease-Good	Benign neoplasm of stomach and duodenum-Good	Oesophagitis and oesophageal ulcer-Good	Irritable bowel syndrome-Good	Cholelithiasis-Not Good	Iron deficiency anaemia-Good	Infections of Other or unspecified organs-Good
41	Diverticular disease of intestine (acute and chronic)	Diverticular disease of intestine (acute and chronic)-Good	Benign neoplasm of colon, rectum, anus and anal canal-Not Good	Peritonitis-Good	Irritable bowel syndrome-Not Good	Abdominal Aortic Aneurysm-Good	Diaphragmatic hernia-Not Good	Anorectal prolapse-Not Good	Benign neoplasm of stomach and duodenum-Not Good	Osteoarthritis (excl spine)-Not Good	Gastritis and duodenitis-Not Good
42	Carpal tunnel syndrome	Carpal tunnel syndrome-Good	Enthesopathies & synovial disorders-Good	Spondylosis-Not Good	Peripheral neuropathies (excluding cranial nerve and carpal tunnel syndromes)-Not Good	Menorrhagia and polymenorrhoea-Not Good	Intervertebral disc disorders-Not Good	Osteoarthritis (excl spine)-Good	Fracture of wrist-Good	Obesity-Good	Fibromatoses-Not Good
43	Seborrheic dermatitis	Seborrheic dermatitis-Good	Dermatitis (atopc/contact/other/unspecified)-Not Good	Rosacea-Not Good	Psoriasis-Not Good	Urticaria-Not Good	Acne-Not Good	Chronic sinusitis-Not Good	Tinnitus-Not Good	Primary Malignancy Other Skin and subcutaneous tissue-Not Good	Enthesopathies & synovial disorders-Not Good
44	Spondylosis	Spondylosis-Good	Intervertebral disc disorders-Good	Osteoarthritis (excl spine)-Good	Spinal stenosis-Good	Carpal tunnel syndrome-Not Good	Spondylolisthesis-Good	Enthesopathies & synovial disorders-Good	Osteoporosis-Good	Transient ischaemic attack-Not Good	Diaphragmatic hernia-Not Good

45	Other or unspecified infectious organisms	Other or unspecified infectious organisms-Good	Bacterial Diseases (excl TB)-Good	Viral diseases (excl chronic hepatitis/HIV)-Good	Infections of Other or unspecified organs-Good	Aspiration pneumonitis-Good	Pleural effusion-Good	Mycoses-Good	Parasitic infections-Good	Pulmonary collapse (excl pneumothorax)-Good	Acute Kidney Injury-Good
46	Vitamin B12 deficiency anaemia	Vitamin B12 deficiency anaemia-Good	Folate deficiency anaemia-Good	Iron deficiency anaemia-Not Good	Other anaemias-Not Good	Coeliac disease-Good	Myelodysplastic syndromes-Good	Dementia-Good	Vitiligo-Good	Rheumatoid Arthritis-Good	Psoriasis-Good
47	Hyperplasia of prostate	Hyperplasia of prostate-Good	Primary Malignancy Prostate-Not Good	Primary Malignancy Bladder-Not Good	Erectile dysfunction-Not Good	Urolithiasis-Good	Hydrocoele (incl infected)-Not Good	Right bundle branch block-Good	Abdominal Hernia-Not Good	Primary Malignancy Other Skin and subcutaneous tissue-Not Good	Diverticular disease of intestine (acute and chronic)-Not Good
48	Iron deficiency anaemia	Iron deficiency anaemia-Good	Other anaemias-Not Good	Folate deficiency anaemia-Not Good	Vitamin B12 deficiency anaemia-Not Good	Menorrhagia and polymenorrhoea-Good	Gastritis and duodenitis-Good	Leiomyoma of uterus-Good	Benign neoplasm of stomach and duodenum-Good	Peptic ulcer disease-Good	Anxiety disorders-Good
49	Female genital prolapse	Female genital prolapse-Good	Anorectal prolapse-Good	Postmenopausal bleeding-Good	Benign neoplasm and polyp of uterus-Good	Urinary Incontinence-Good	Neuromuscular dysfunction of bladder-Good	Postcoital and contact bleeding-Good	Sjogren's disease-Not Good	Leiomyoma of uterus-Good	Primary Malignancy Breast-Not Good
50	Urinary Incontinence	Urinary Incontinence-Good	Neuromuscular dysfunction of bladder-Good	Female genital prolapse-Good	Hearing loss-Good	Depression-Good	Osteoarthritis (excl spine)-Good	Allergic and chronic rhinitis-Good	Dementia-Good	Enthesopathies & synovial disorders-Not Good	Obesity-Good
51	Other anaemias	Other anaemias-Good	Iron deficiency anaemia-Good	Folate deficiency anaemia-Good	Depression-Good	Vitamin B12 deficiency anaemia-Good	Oesophagitis and oesophageal ulcer-Good	Dermatitis (atopc/contact /other/unspecified)-Good	Peptic ulcer disease-Good	Heart failure-Good	Primary Malignancy colorectal and anus-Good

52	Diabetic ophthalmic complications	Diabetic ophthalmic complications-Good	Diabetic neurological complications-Good	Type 1 Diabetes Mellitus, Type 2 Diabetes Mellitus, and Diabetes Mellitus ‚Äì other or not specified-Good	Macular degeneration-Good	Erectile dysfunction-Good	Retinal vascular occlusions-Good	Visual impairment and blindness-Good	Glaucoma-Good	Obesity-Good	Retinal detachment-Good
53	Heart failure	Heart failure-Good	Other Cardiomyopathy-Good	Nonrheumatic mitral valve disorders-Good	Left bundle branch block-Good	Myocardial Infarction-Good	Nonrheumatic aortic valve disorders-Good	Dilated cardiomyopathy-Good	Coronary heart disease not otherwise specified-Good	Atrioventricular block, complete-Good	Primary pulmonary hypertension-Not Good
54	Stroke Not otherwise specified (NOS)	Stroke Not otherwise specified (NOS)-Good	Ischaemic stroke-Good	Transient ischaemic attack-Good	Intracerebral haemorrhage-Good	Aspiration pneumonitis-Good	Dementia-Good	Epilepsy-Good	Subarachnoid haemorrhage-Good	Heart failure-Good	Delirium, not induced by alcohol and other psychoactive substances-Good
55	Irritable bowel syndrome	Irritable bowel syndrome-Good	Chronic sinusitis-Good	Diverticular disease of intestine (acute and chronic)-Not Good	Anxiety disorders-Good	Allergic and chronic rhinitis-Good	Anal fissure-Not Good	Migraine-Good	Gastritis and duodenitis-Not Good	Dysmenorrhoea-Good	Gastro-oesophageal reflux disease-Not Good

56	Gout	Gout-Good	Type 1 Diabetes Mellitus, Type 2 Diabetes Mellitus, and Diabetes Mellitus ‚Äì other or not specified-Good	Primary Malignancy Other Skin and subcutaneous tissue-Good	Osteoarthritis (excl spine)-Good	Heart failure-Good	Glomerulonephritis-Good	Erectile dysfunction-Good	Actinic keratosis-Not Good	Enthesopathies & synovial disorders-Not Good	Fibromatoses-Not Good
57	Oesophagitis and oesophageal ulcer	Oesophagitis and oesophageal ulcer-Good	Diaphragmatic hernia-Not Good	Barrett's oesophagus-Good	Gastro-oesophageal reflux disease-Good	Gastritis and duodenitis-Good	Peptic ulcer disease-Good	Benign neoplasm of stomach and duodenum-Good	Other anaemias-Good	Depression-Good	Lower Respiratory Tract Infections-Good
58	Erectile dysfunction	Erectile dysfunction-Good	Type 1 Diabetes Mellitus, Type 2 Diabetes Mellitus, and Diabetes Mellitus ‚Äì other or not specified-Good	Hyperplasia of prostate-Not Good	Primary Malignancy Prostate-Good	Diabetic ophthalmic complications-Good	Dyslipidaemia-Good	Peripheral neuropathies (excluding cranial nerve and carpal tunnel syndromes)-Good	Sleep apnoea-Good	Fibromatoses-Good	Abdominal Hernia-Not Good
59	Dementia	Dementia-Good	Delirium, not induced by alcohol and other psychoactive substances-Not Good	Parkinson's disease-Good	Aspiration pneumonitis-Good	Fracture of hip-Good	Left bundle branch block-Good	End stage renal disease-Good	Stroke Not otherwise specified (NOS)-Good	Ischaemic stroke-Good	Intracerebral haemorrhage-Good

60	Alcohol Problems	Alcohol Problems-Good	Liver fibrosis, sclerosis and cirrhosis-Good	Hepatic failure-Good	Alcoholic liver disease-Good	Fatty Liver-Good	Other psychoactive substance misuse-Good	Portal hypertension-Good	Pancreatitis-Good	Oesophageal varices-Good	Personality disorders-Good
61	Other psychoactive substance misuse	Other psychoactive substance misuse-Good	Chronic viral hepatitis-Good	Personality disorders-Good	Schizophrenia, schizotypal and delusional disorders-Not Good	Obsessive-compulsive disorder-Good	Anorexia and bulimia nervosa-Not Good	Bipolar affective disorder and mania-Good	Alcohol Problems-Good	Alopecia areata-Not Good	Crohn's disease-Not Good
62	Acne	Acne-Good	Rosacea-Not Good	Dysmenorrhoea-Good	Postcoital and contact bleeding-Not Good	Carcinoma in situ cervical-Not Good	Polycystic ovarian syndrome-Good	Hidradenitis suppurativa-Not Good	Prematurity-Not Good	Menorrhagia and polymenorrhoea-Not Good	Crohn's disease-Good
63	Psoriasis	Psoriasis-Good	Psoriatic arthropathy-Good	Seborrheic dermatitis-Not Good	Rheumatoid Arthritis-Good	Dermatitis (atopc/contact/other/unspecified)-Good	Obesity-Good	Hidradenitis suppurativa-Good	Urticaria-Not Good	Gastro-oesophageal reflux disease-Not Good	Bipolar affective disorder and mania-Not Good
64	Chronic sinusitis	Chronic sinusitis-Good	Allergic and chronic rhinitis-Good	Hypertrophy of nasal turbinates-Good	Irritable bowel syndrome-Good	Nasal polyp-Good	Asthma-Good	Migraine-Not Good	Seborrheic dermatitis-Not Good	Dysmenorrhoea-Not Good	Urticaria-Good
65	Abdominal Hernia	Abdominal Hernia-Good	Hydrocoele (incl infected)-Good	Erectile dysfunction-Not Good	Hyperplasia of prostate-Good	Appendicitis-Not Good	Primary Malignancy Other Skin and subcutaneous tissue-Not Good	Hearing loss-Not Good	Actinic keratosis-Not Good	Enthesopathies & synovial disorders-Not Good	Psoriasis-Not Good

66	Obesity	Obesity-Good	Type 1 Diabetes Mellitus, Type 2 Diabetes Mellitus, and Diabetes Mellitus ‚Äì other or not specified-Good	Sleep apnoea-Good	Polycystic ovarian syndrome-Good	Dyslipidaemia-Good	Hidradenitis suppurativa-Good	Menorrhagia and polymenorrhoea-Good	Diabetic ophthalmic complications-Good	Allergic and chronic rhinitis-Good	Osteoarthritis (excl spine)-Good
67	Hearing loss	Hearing loss-Good	Tinnitus-Good	Enthesopathies & synovial disorders-Not Good	Osteoarthritis (excl spine)-Good	Urinary Incontinence-Good	Dermatitis (atopc/contact/other/unspecified)-Not Good	Allergic and chronic rhinitis-Good	Visual impairment and blindness-Not Good	Actinic keratosis-Not Good	Seborrheic dermatitis-Good
68	Migraine	Migraine-Good	Menorrhagia and polymenorrhoea-Good	Dysmenorrhoea-Good	Irritable bowel syndrome-Good	Chronic sinusitis-Not Good	Acne-Good	Postviral fatigue syndrome, neurasthenia and fibromyalgia-Not Good	Asthma-Good	Enthesopathies & synovial disorders-Not Good	Allergic and chronic rhinitis-Not Good
69	Menorrhagia and polymenorrhoea	Menorrhagia and polymenorrhoea-Good	Leiomyoma of uterus-Good	Dysmenorrhoea-Good	Postcoital and contact bleeding-Good	Endometrial hyperplasia and hypertrophy-Good	Endometriosis-Good	Benign neoplasm and polyp of uterus-Good	Carcinoma in situ cervical-Good	Migraine-Good	Benign neoplasm of ovary-Good

70	Type 1 Diabetes Mellitus, Type 2 Diabetes Mellitus, and Diabetes Mellitus ‚Äì other or not specified	Type 1 Diabetes Mellitus, Type 2 Diabetes Mellitus, and Diabetes Mellitus ‚Äì other or not specified-Good	Diabetic ophthalmic complications-Good	Dyslipidaemia-Good	Erectile dysfunction-Good	Obesity-Good	Diabetic neurological complications-Good	Peripheral arterial disease-Good	Gout-Good	Hypertension-Good	Osteoarthritis (excl spine)-Good
71	Cataract	Cataract-Good	Macular degeneration-Good	Glaucoma-Good	Visual impairment and blindness-Good	Retinal detachment-Good	Anterior and Intermediate Uveitis-Good	Diabetic ophthalmic complications-Good	Atrial Fibrillation and flutter-Not Good	Osteoporosis-Good	Hearing loss-Good
72	Stable Angina	Stable Angina-Good	Unstable Angina-Good	Myocardial Infarction-Good	Coronary heart disease not otherwise specified-Good	Dyslipidaemia-Good	Heart failure-Good	Peripheral arterial disease-Good	Nonrheumatic aortic valve disorders-Good	Transient ischaemic attack-Good	Left bundle branch block-Good
73	Gastro-oesophageal reflux disease	Gastro-oesophageal reflux disease-Good	Diaphragmatic hernia-Good	Gastritis and duodenitis-Good	Oesophagitis and oesophageal ulcer-Good	Anxiety disorders-Good	Barrett's oesophagus-Good	Irritable bowel syndrome-Good	Benign neoplasm of stomach and duodenum-Good	Allergic and chronic rhinitis-Good	Chronic sinusitis-Not Good
74	Coronary heart disease not otherwise specified	Coronary heart disease not otherwise specified-Good	Myocardial Infarction-Good	Stable Angina-Good	Unstable Angina-Good	Heart failure-Good	Atrial Fibrillation and flutter-Good	Left bundle branch block-Good	Dyslipidaemia-Good	Peripheral arterial disease-Good	Atrioventricular block, first degree-Good

75	Chronic obstructive pulmonary disease (COPD)	Chronic obstructive pulmonary disease (COPD)-Good	Asthma-Not Good	Respiratory failure-Good	Lower Respiratory Tract Infections-Not Good	Bronchiectasis-Not Good	Pneumothorax-Good	Primary Malignancy Lung and trachea-Good	Heart failure-Good	Other interstitial pulmonary diseases with fibrosis-Not Good	Abdominal Aortic Aneurysm-Good
76	Dyslipidaemia	Dyslipidaemia-Good	Type 1 Diabetes Mellitus, Type 2 Diabetes Mellitus, and Diabetes Mellitus ‚Äì other or not specified-Good	Enthesopathies & synovial disorders-Not Good	Osteoarthritis (excl spine)-Good	Stable Angina-Good	Coronary heart disease not otherwise specified-Good	Obesity-Good	Hypertension-Good	Erectile dysfunction-Good	Allergic and chronic rhinitis-Good
77	Epilepsy	Epilepsy-Good	Intellectual disability-Good	Autism and Asperger's syndrome-Good	Primary Malignancy Brain, Other CNS and Intracranial-Good	Intracerebral haemorrhage-Good	Stroke Not otherwise specified (NOS)-Not Good	Aspiration pneumonitis-Good	Other psychoactive substance misuse-Good	Subarachnoid haemorrhage-Good	Alcohol Problems-Good
78	Hypo or hyperthyroidism	Hypo or hyperthyroidism-Good	Hypertension-Good	Primary Malignancy Thyroid-Good	Atrial Fibrillation and flutter-Good	Dyslipidaemia-Good	Dermatitis (atopc/contact/other/unspecified)-Good	Osteoarthritis (excl spine)-Not Good	Benign neoplasm of brain and other parts of central nervous system-Good	Obesity-Good	Asthma-Good
79	Allergic and chronic rhinitis	Allergic and chronic rhinitis-Good	Chronic sinusitis-Good	Dermatitis (atopc/contact/other/unspecified)-Good	Asthma-Good	Enthesopathies & synovial disorders-Not Good	Irritable bowel syndrome-Good	Nasal polyp-Good	Osteoarthritis (excl spine)-Not Good	Hypertrophy of nasal turbinates-Good	Hearing loss-Good

80	Atrial Fibrillation and flutter	Atrial Fibrillation and flutter-Good	Pleural effusion-Not Good	Coronary heart disease not otherwise specified-Good	Supraventricular tachycardia-Good	Heart failure-Good	Hypertension-Not Good	Primary pulmonary hypertension-Good	Cataract-Good	Nonrheumatic mitral valve disorders-Good	Nonrheumatic aortic valve disorders-Not Good
81	Osteoarthritis (excl spine)	Osteoarthritis (excl spine)-Good	Enthesopathies & synovial disorders-Good	Spondylosis-Good	Dermatitis (atopc/contact/other/unspecified)-Not Good	Hearing loss-Good	Dyslipidaemia-Good	Allergic and chronic rhinitis-Not Good	Hypertension-Good	Diverticular disease of intestine (acute and chronic)-Not Good	Gastro-oesophageal reflux disease-Good
82	Asthma	Asthma-Good	Chronic obstructive pulmonary disease (COPD)-Not Good	Allergic and chronic rhinitis-Good	Chronic sinusitis-Good	Dermatitis (atopc/contact/other/unspecified)-Good	Nasal polyp-Good	Migraine-Good	Enthesopathies & synovial disorders-Not Good	Obesity-Good	Menorrhagia and polymenorrhoea-Not Good
83	Anxiety disorders	Anxiety disorders-Good	Depression-Good	Gastro-oesophageal reflux disease-Good	Irritable bowel syndrome-Good	Dermatitis (atopc/contact/other/unspecified)-Good	Chronic sinusitis-Good	Allergic and chronic rhinitis-Good	Iron deficiency anaemia-Good	Other psychoactive substance misuse-Good	Enthesopathies & synovial disorders-Not Good
84	Enthesopathies & synovial disorders	Enthesopathies & synovial disorders-Good	Osteoarthritis (excl spine)-Good	Dermatitis (atopc/contact/other/unspecified)-Not Good	Allergic and chronic rhinitis-Not Good	Carpal tunnel syndrome-Not Good	Hearing loss-Not Good	Dyslipidaemia-Good	Depression-Not Good	Spondylosis-Good	Migraine-Not Good
85	Dermatitis (atopc/contact/other/unspecified)	Dermatitis (atopc/contact/other/unspecified)-Good	Seborrheic dermatitis-Good	Enthesopathies & synovial disorders-Not Good	Allergic and chronic rhinitis-Good	Osteoarthritis (excl spine)-Not Good	Urticaria-Good	Depression-Good	Hypertension-Good	Asthma-Good	Hearing loss-Good
86	Depression	Depression-Good	Anxiety disorders-Good	Dermatitis (atopc/contact/other/unspecified)-Good	Other anaemias-Good	Enthesopathies & synovial disorders-Not Good	Chronic sinusitis-Good	Irritable bowel syndrome-Good	Allergic and chronic rhinitis-Good	Osteoarthritis (excl spine)-Good	Urinary Incontinence-Good

87	Hypertension	Hypertension-Good	Dermatitis (atopc/contact/other/unspecified)-Good	Osteoarthritis (excl spine)-Good	Atrial Fibrillation and flutter-Good	Dyslipidaemia-Good	Type 1 Diabetes Mellitus, Type 2 Diabetes Mellitus, and Diabetes Mellitus ‚Äì other or not specified-Good	Enthesopathies & synovial disorders-Not Good	Depression-Good	Hypo or hyperthyroidism-Good	Acute Kidney Injury-Good

Hyperparameter Tuning for RETAIN and Deepr

In this section, we performed Bayesian Optimisation to search for optimal parameters for Deepr (Table S4) and RETAIN (Table S5).

Table S4. Deepr Best Model for Subsequent Visit Prediction Task

Iteration	Filters	Kernel Size	FC I	FC II	FC III	Dropout I	Dropout II	Dropout III	Learning Rate	Average Precision
1	37	7	10	46	28	0.4139	0.4997	0.3718	0.0004	0.2599
2	24	5	28	19	13	0.4936	0.1722	0.4643	0.0062	0.2319
3	17	7	16	48	40	0.2250	0.3945	0.3264	0.0310	0.1815
4	33	7	6	10	25	0.1602	0.4476	0.3544	0.0026	0.2640
5	32	7	4	30	32	0.3714	0.3382	0.3573	0.0353	0.2005
6	13	4	50	17	47	0.3424	0.1830	0.3056	0.0008	0.3274
7	12	3	3	6	43	0.4201	0.2387	0.3884	0.0123	0.2112
8	30	7	16	26	41	0.2055	0.3343	0.1541	0.0011	0.3256
9	35	5	50	24	25	0.1567	0.4056	0.1329	0.0004	0.3433
10	41	7	9	35	19	0.4885	0.3548	0.3080	0.0004	0.2343
11	4	4	33	12	39	0.1811	0.1251	0.1066	0.0010	0.3051
12	48	4	36	45	37	0.2143	0.3486	0.1222	0.0015	0.3504
13	36	3	39	10	48	0.1992	0.4164	0.1183	0.0050	0.3291
14	45	4	44	40	26	0.2329	0.2900	0.1280	0.0903	0.0042
15	40	4	35	48	11	0.1600	0.4704	0.1211	0.0019	0.3125
**16**	**49**	**3**	**47**	**41**	**40**	**0.1002**	**0.2541**	**0.1284**	**0.0019**	**0.3588**
17	47	3	50	47	12	0.2519	0.2125	0.1668	0.0005	0.2988
18	47	3	37	35	50	0.1059	0.4225	0.1120	0.0034	0.3487
19	47	4	48	34	39	0.2177	0.3607	0.1301	0.0016	0.3567
20	46	3	46	45	43	0.2007	0.1609	0.1196	0.0044	0.3356

Table S5. RETAIN Best Model for Subsequent Visit Prediction Task

Iteration	Embedding Size	Recurrent Size	Dropout Embedding	Dropout Context	L2	Average Precision
1	142	90	0.3846	0.0224	0.0891	0.1822
2	124	43	0.3922	0.0382	0.0003	0.1815
3	173	90	0.3929	0.2238	0.0014	0.3479
4	145	91	0.4117	0.0404	0.0102	0.2049
5	153	92	0.4569	0.0642	0.0116	0.2049
6	120	37	0.4335	0.4017	0.0728	0.1815
7	180	102	0.3567	0.3307	0.0039	0.2469
**8**	**195**	**38**	**0.3805**	**0.2711**	**0.0010**	**0.3740**
9	165	119	0.4969	0.1964	0.0047	0.2292
10	174	92	0.3862	0.2078	0.0019	0.3329
11	145	110	0.3928	0.1926	0.0891	0.1813
12	195	83	0.4418	0.4787	0.0011	0.3543
13	187	110	0.3456	0.2083	0.0123	0.2122
14	144	80	0.3717	0.0932	0.0032	0.3038
15	193	68	0.4528	0.3950	0.0022	0.3280
16	198	45	0.4344	0.3324	0.0442	0.1828
17	145	64	0.4213	0.1950	0.0626	0.1813
18	171	116	0.4166	0.4950	0.0028	0.3197
19	186	38	0.4062	0.4916	0.0011	0.3309
20	136	54	0.3503	0.1678	0.0067	0.2162

Disease-wise Model Performance

In this section, we show the disease-wise BEHRT performance in terms of AUROC and APS for the next 6 months prediction task. We have displayed codes with occurrence ratio 0.01 or higher. And detailed below is the description of the Caliber code and the chapter along with the APS/AUROC.

Table S6. Diseases Prediction Performance

Caliber	APS	AUROC	Description	Ratio	Caliber Chapter
92	0.066765	0.723828	Gastritis and duodenitis	0.011198	Diseases of the digestive system
66	0.108185	0.797633	Diaphragmatic hernia	0.011490	Diseases of the digestive system
100	0.118093	0.742646	Hearing loss	0.021964	Diseases of the ear and mastoid process
273	0.132567	0.773249	Spondylosis	0.013459	Diseases of the musculoskeletal system and connective tissue
189	0.142594	0.844596	Pleural effusion	0.010229	Diseases of the respiratory system
172	0.162186	0.798654	Other anaemias	0.023303	Diseases of the blood and blood-forming organs and certain disorders involving the immune mechanism
29	0.163355	0.822707	Bacterial Diseases (excl TB)	0.023979	Certain infectious and parasitic diseases
130	0.167457	0.804545	Iron deficiency anaemia	0.020780	Diseases of the blood and blood-forming organs and certain disorders involving the immune mechanism
295	0.169674	0.836610	Urinary Tract Infections	0.022534	Diseases of the genitourinary system

Table S6. Diseases Prediction Performance

69	0.170852	0.811759	Diverticular disease of intestine (acute and chronic)	0.015966	Diseases of the digestive system
8	0.177170	0.810068	Allergic and chronic rhinitis	0.023241	Diseases of the respiratory system
170	0.181182	0.847938	Osteoporosis	0.013982	Diseases of the musculoskeletal system and connective tissue
71	0.184000	0.790655	Dyslipidaemia	0.026010	Endocrine, nutritional and metabolic diseases
168	0.184629	0.799592	Oesophagitis and oesophageal ulcer	0.022426	Diseases of the digestive system
217	0.203218	0.855593	Primary Malignancy Other Skin and subcutaneous tissue	0.012013	Neoplasms
93	0.206455	0.776367	Gastro-oesophageal reflux disease	0.026271	Diseases of the digestive system
290	0.208524	0.814481	Type 1 Diabetes Mellitus, Type 2 Diabetes Mellitus, and Diabetes Mellitus – other or not specified	0.021226	Endocrine, nutritional and metabolic diseases
3	0.219637	0.869366	Actinic keratosis	0.012490	Diseases of the skin and subcutaneous tissue
131	0.220017	0.874319	Irritable bowel syndrome	0.011182	Diseases of the digestive system
294	0.223863	0.824114	Urinary Incontinence	0.020057	Diseases of the genitourinary system
169	0.234444	0.785766	Osteoarthritis (excl spine)	0.043714	Diseases of the musculoskeletal system and connective tissue
176	0.245906	0.834062	Other or unspecified infectious organisms	0.030963	Diseases of the respiratory system
95	0.249208	0.894444	Glaucoma	0.011367	Diseases of the eye and adnexa
109	0.250573	0.885547	Hyperplasia of prostate	0.020842	Diseases of the genitourinary system
184	0.264687	0.879325	Peripheral arterial disease	0.010951	Diseases of the circulatory system
79	0.265863	0.762939	Enthesopathies & synovial disorders	0.047036	Diseases of the musculoskeletal system and connective tissue
81	0.267187	0.905812	Erectile dysfunction	0.017873	Mental and behavioural disorders
140	0.268504	0.867094	Lower Respiratory Tract Infections	0.023518	Certain infectious and parasitic diseases
63	0.271027	0.753816	Dermatitis (atopc/contact/other/unspecified)	0.049051	Diseases of the skin and subcutaneous tissue
142	0.292598	0.893802	Macular degeneration	0.010752	Diseases of the eye and adnexa
274	0.296798	0.889236	Stable Angina	0.032039	Diseases of the circulatory system
57	0.301041	0.900088	Coronary heart disease not otherwise specified	0.035177	Diseases of the circulatory system
275	0.307238	0.911618	Stroke Not otherwise specified (NOS)	0.023395	Diseases of the nervous system
45	0.319433	0.863447	Cataract	0.042099	Diseases of the eye and adnexa
1	0.319972	0.845171	Abdominal Hernia	0.019180	Diseases of the digestive system
44	0.325143	0.843480	Carpal tunnel syndrome	0.012013	Diseases of the nervous system
101	0.334902	0.912117	Heart failure	0.024918	Diseases of the circulatory system
164	0.335131	0.879670	Obesity	0.017442	Endocrine, nutritional and metabolic diseases

Table S6. Diseases Prediction Performance

22	0.348523	0.885055	Asthma	0.026133	Diseases of the respiratory system
97	0.349361	0.882694	Gout	0.018058	Diseases of the musculoskeletal system and connective tissue
65	0.350132	0.942604	Diabetic ophthalmic complications	0.018919	Endocrine, nutritional and metabolic diseases
147	0.368465	0.911541	Migraine	0.012028	Diseases of the nervous system
229	0.398842	0.904686	Psoriasis	0.011751	Diseases of the musculoskeletal system and connective tissue
17	0.410899	0.858498	Anxiety disorders	0.041914	Mental and behavioural disorders
146	0.433645	0.969406	Menorrhagia and polymenorrhoea	0.015504	Diseases of the genitourinary system
113	0.489456	0.905032	Hypo or hyperthyroidism	0.047897	Endocrine, nutritional and metabolic diseases
302	0.491672	0.855823	Vitamin B12 deficiency anaemia	0.014489	Diseases of the blood and blood-forming organs and certain disorders involving the immune mechanism
51	0.501496	0.923082	Chronic obstructive pulmonary disease (COPD)	0.036869	Diseases of the respiratory system
23	0.514881	0.901268	Atrial Fibrillation and flutter	0.077629	Diseases of the circulatory system
110	0.531597	0.819527	Hypertension	0.200618	Diseases of the circulatory system
61	0.542223	0.950442	Dementia	0.024656	Mental and behavioural disorders
62	0.553561	0.877904	Depression	0.076876	Mental and behavioural disorders
85	0.573880	0.934049	Female genital prolapse	0.015781	Diseases of the genitourinary system
220	0.575574	0.964776	Primary Malignancy Prostate	0.011844	Neoplasms
6	0.583305	0.952656	Alcohol Problems	0.014535	Mental and behavioural disorders
194	0.647243	0.955062	Polymyalgia Rheumatica	0.013213	Diseases of the musculoskeletal system and connective tissue
80	0.648763	0.977907	Epilepsy	0.016104	Diseases of the nervous system

Comparison of APS/AUROC across Models

Figure S1, shows the performance three models, in terms of AUROC and APS, for each and every disease with a prevalence of 1% or higher. BEHRT's predictions remain in the upper right quadrant of the graph (for the most part) demonstrating a higher APS/AUROC than the other two models.

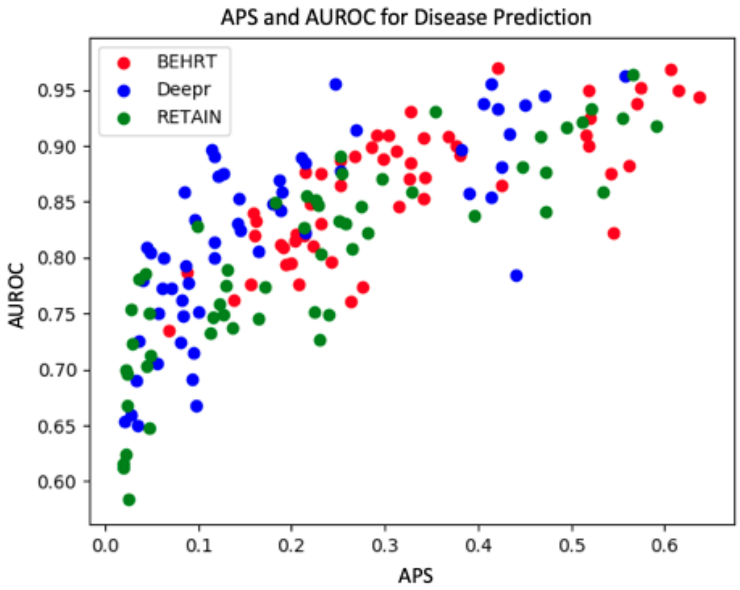

Figure S1. Disease-wise performance comparison for BEHRT, Deepr and RETAIN, all models trained on same dataset and trained on the same task (next 6 months), each point represents the APS/AUROC of one disease on corresponding model.

Gender-Specific Disease Analysis

Table S7 illustrates the predictive performance of the model for gender-specific diseases. Male and female predictions in the table represent the number of patients with predictive probability above 0.5 for that gender, and disease gender indicates whether a disease is a male-specific or female-specific disease. M and F represent male and female respectively.

Table S7. Predictions of Gender-Specific Diseases (Test Predictions from Next 6 Months)

Disease Name	Disease Gender	Male Predictions	Female Predictions
Hyperplasia of Prostate	M	384	0
Hydrocoele (incl infected)	M	36	0
Male Infertility	M	1	24
Primary Malignancy Prostate	M	557	0
Erectile Dysfunction	M	425	1
Menorrhagia and Polymenorrhoea	F	0	697
Endometriosis	F	0	47
Female Genital Prolapse	F	0	865
Female Infertility	F	2	36
Benign Neoplasm of Ovary	F	0	69
Postmenopausal Bleeding	F	0	140
Primary Malignancy Breast	F	0	11
Primary Malignancy Ovarian	F	1	193

Male (M) and Female (F) Predictions imply label predictions >0.5

Feature importance comparison

Table S8 illustrates an ablation study to selectively remove features (age, segment and position) to understand the importance of each feature.

Table S8. Feature Importance Comparison for the Next 6 Months Prediction Task

Features				Metrics	
Disease	Age	Segment	Position	APS	AUROC
1	1	1	1	0.525	0.958
1	0	1	1	0.515	0.954
1	1	0	1	0.511	0.954
1	1	1	0	0.498	0.954
1	0	1	0	0.451	0.952
1	1	0	0	0.501	0.955
1	0	0	0	0.446	0.951

1: Features activated, 0: Features deactivated
